# Effect of evolocumab on the progression of intraplaque neovascularization of the carotid based on contrast-enhanced ultrasonography (EPIC study): A prospective single-arm, open-label study

**DOI:** 10.3389/fphar.2022.999224

**Published:** 2023-01-04

**Authors:** Ju Chen, Faming Zhao, Chengbin Lei, Tianjun Qi, Xin Xue, Yuan Meng, Wenzhong Zhang, Hui Zhang, Jian Wang, Haijun Zhu, Cheng Cheng, Qilei Wang, Chenglong Bi, Beibei Song, Chengwei Jin, Qiang Niu, Fengshuang An, Bin Li, Xiaoguang Huo, Yunhe Zhao, Bo Li

**Affiliations:** ^1^ Department of Medical Ultrasonics, Zibo Central Hospital, Zibo, China; ^2^ Department of Cardiology, Zibo Central Hospital, Zibo, China; ^3^ Department of Infectious Disease, Zibo Infectious Disease Hospital, Zibo, China; ^4^ Laboratory Department, Zibo Central Hospital, Zibo, China; ^5^ Department of Cardiology, Qilu Hospital of Shandong University, Jinan, China; ^6^ Department of Cardiology, Central Hospital Affiliated to Shandong First Medical University, Jinan, Shandong, China

**Keywords:** proprotein convertase subtilisin-kexin type 9, evolocumab, atherosclerotic cardiovascular disease, intra-plaque neovascularization, contrast-enhanced ultrasonography

## Abstract

**Background and Purpose:** The aim of this study was to explore the effect of half a year of evolocumab plus moderate-intensity statin treatment on carotid intraplaque neovascularization (IPN) and blood lipid levels.

**Methods:** A total of 31 patients with 33 carotid plaques who received evolocumab plus statin treatment were included. Blood lipid levels, B-mode ultrasound and contrast-enhanced ultrasonography (CEUS) at baseline and after half a year of evolocumab plus statin therapy were collected. The area under the curve (AUC) reflected the total amount of acoustic developer entering the plaque or lumen within the 180 s measurement period. The enhanced intensity reflected the peak blood flow intensity during the monitoring period, and the contrast agent area reflected the area of vessels in the plaques.

**Results:** Except for high-density lipoprotein cholesterol (HDL-c), all other lipid indices decreased. Compared with baseline, low-density lipoprotein cholesterol (LDL-c) decreased by approximately 57% (*p* < 0.001); total cholesterol (TC) decreased by approximately 34% (*p* < 0.001); small dense low-density lipoprotein (sd-LDL) decreased by approximately 52% (*p* < 0.001); and HDL-c increased by approximately 20% (*p* < 0.001). B-mode ultrasonography showed that the length and thickness of the plaque and the hypoechoic area ratio were reduced (*p* < 0.05). The plaque area, calcified area ratio, and lumen cross-sectional area changed little (*p* > 0.05). CEUS revealed that the area under the curve of plaque/lumen [AUC (P/L)] decreased from 0.27 ± 0.13 to 0.19 ± 0.11 (*p* < 0.001). The enhanced intensity ratio of plaque/lumen [intensity ratio (P/L)] decreased from 0.37 ± 0.16 to 0.31 ± 0.14 (*p* = 0.009). The contrast agent area in plaque/area of plaque decreased from 19.20 ± 13.23 to 12.66 ± 9.59 (*p* = 0.003). The neovascularization score decreased from 2.64 ± 0.54 to 2.06 ± 0.86 (*p* < 0.001). Subgroup analysis based on statin duration (<6 months and ≥6 months) showed that there was no significant difference in the AUC (P/L) or intensity ratio (P/L) at baseline or after half a year of evolocumab treatment.

**Conclusion:** This study found that evolocumab combined with moderate-intensity statins significantly improved the blood lipid profile and reduced carotid IPN.

**Clinical Trial Registration:** https://www.clinicaltrials.gov; identifier: NCT04423406.

## Introduction

Evolocumab is a novel lipid-lowering drug that can specifically inhibit proprotein convertase subtilisin-kexin type 9 (PCSK9), an enzyme that regulates serum low-density lipoprotein cholesterol (LDL-c) levels through the degradation of the LDL receptor (Yurtseven et al., 2020; Mercep et al., 2022). According to recent studies, blocking PCSK9 with specific inhibitors causes a significant decrease in LDL-c levels with or without statin cotreatment (Landmesser et al., 2017; Reyes-Soffer et al., 2017; Sabatine et al., 2017; Diaz et al., 2021; Sever et al., 2021). The FOURIER trial (Further Cardiovascular Outcomes Research with PCSK9 Inhibition in Subjects with Elevated Risk) showed that evolocumab lowered LDL-c levels to a median of 0.78 mmol/L and reduced the risk of cardiovascular events by 15% (Sabatine et al., 2017). Another study showed that lower LDL-c was related to more stable plaque (Kataoka et al., 2015). The Global Assessment of Plaque Regression with a PCSK9 Antibody as Measured by Intravascular Ultrasound (GLAGOV) study found that if LDL-c is low enough, it can even reverse atherosclerotic plaque (Nicholls et al., 2016). Based on these findings, we hypothesized that carotid plaques would become more stable with evolocumab treatment.

Plaque stability depends on several factors, including lipid content, thickness of the fibrous cap, and inflammatory reactions (Hafiane, 2019). In addition, angiogenesis plays a crucial role in determining plaque development and vulnerability. Several *in vivo* studies have demonstrated that increased microvessels are associated with plaque rupture (Dunmore et al., 2007; Kodama et al., 2012). Furthermore, vessel density was found to be two times greater in vulnerable plaques than in stable plaques and four times greater in ruptured plaques than in stable plaques (Chen et al., 2013). Therefore, identifying intraplaque neovascularization (IPN) has great significance for identifying vulnerable plaques. Magnetic resonance angiography (MRA) is the current gold standard to identify IPN in plaques with high resolution and sensitivity, but its high cost and long processing time limit its use. Traditional ultrasonography is a cost-effective carotid plaque screening imaging modality used in China, but the resolution of color/power Doppler is not sufficient for identifying IPN. However, contrast-enhanced ultrasonography (CEUS) may allow increased dynamic visualization of IPN distribution and density, providing a comprehensive assessment of plaque stability (Cui et al., 2021; Huang et al., 2021; Lyu et al., 2021).

To our knowledge, this is the first study to use CEUS for carotid IPN detection and carotid plaque stability evaluation after evolocumab treatment combined with statin therapy.

## Patients and methods

### Patients

This prospective, single-center, open-label, single-arm, comparative study was approved by our Research Ethics Committee. The study was explained to the participants by the investigator, and all participants signed written informed consent forms. From August 2020 to July 2021, 32 patients who met all of the following criteria were recruited: ① there was at least one carotid atherosclerotic plaque on ultrasound imaging, defined according to the Mannheim consensus as the presence of focal structures encroaching into the arterial lumen by > 0.5 mm, by 50% of the thickness of the surrounding intima-media complex, or by the thickness of the intima-media layer if this was >1.5 mm (Touboul et al., 2012); ② plaques thicker than 1.5 mm and uniformly or predominantly echolucent as determined by B-mode ultrasonography; ③ plaques had IPN determined by CEUS; ④ patients had been treated with statins for at least 1 month before enrollment, with LDL-c higher than 1.8 mmol/L and triglyceride (TG) lower than 4.5 mmol/L. Patients were excluded if they had known allergies to albumin or SonoVue; were pregnant; had severe liver, kidney or thyroid dysfunction; had severe arrhythmia, heart failure or hypertension; had poor blood glucose control; or had used evolocumab before.

**FIGURE 1:**
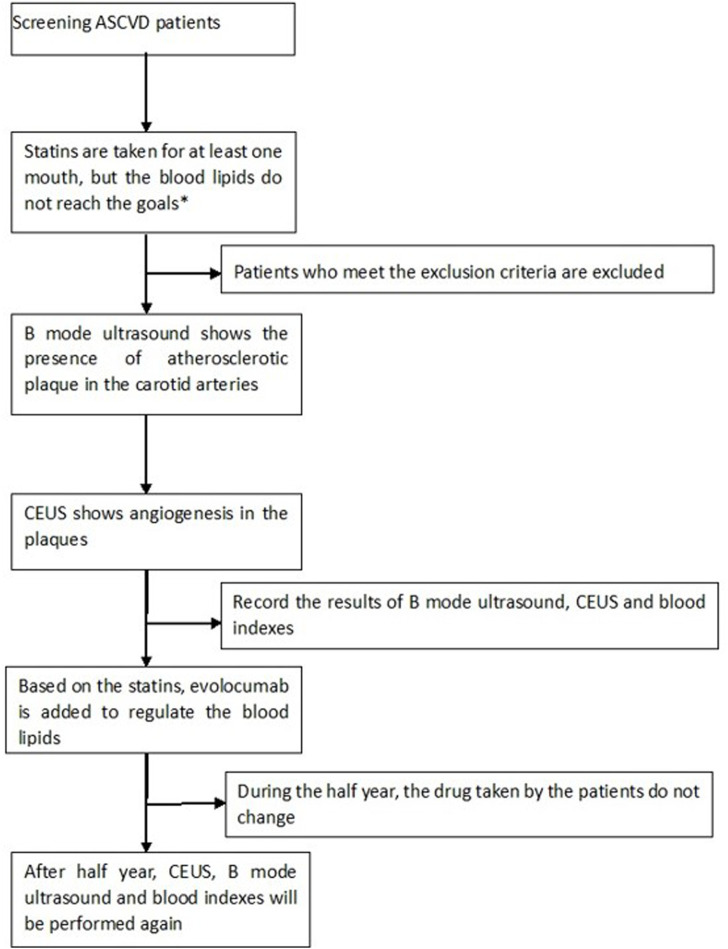
Our target goal for blood lipids was LDL-c<1.8 mmol/L; CEUS: contrast-enhanced ultrasound; ASCVD: arteriosclerotic cardiovascular disease; exclusion criteria: allergies to albumin or SonoVue; pregnancy; severe liver, kidney or thyroid dysfunction; severe arrhythmia, heart failure or hypertension; and diabetes patients with poor control of blood glucose who had used evolocumab before.

Among the 32 patients, 31 completed the follow-up and were included in the analysis; one patient was lost to follow-up. Figure 1 shows the flow chart of this study.

All included patients first underwent moderate-intensity statin therapy: rosuvastatin (10 mg qd po) or an equivalent dose of another statin for at least 1 month, followed by evolocumab (140 mg/2 weeks, ih). Table 1 shows the patients’ demographic characteristics, including sex, age, and comorbidities.

**TABLE 1:** Clinical characteristics of the patients.

Characteristics	Number (n = 31)
Age (years)	58.74 ± 9.19
Gender	
Male	20
Female	11
Smoking	6
Clinical history	
Diabetes	11
Hypertension	20
Stroke	3
Peripheral arterial disease	2
Coronary artery disease	28
Drug use	
Aspirin	31
Clopidogrel	12
Ticagrelor	13
Statins	31
β-blocker	13
Metformin	3
Insulin	3
ARB	7
SGLT-2i	2
GLP-1	1
Number of stent per one patient	1.13
Number of patients with coronary stent implantation	21

Age is presented as the mean ± SD.; ARB, angiotensin II receptor blocker; SGLT-2i, sodium-dependent glucose transporters 2 inhibitor; GLP-1, glucagon-like peptide-1.

### Analysis of blood lipid levels

A total of 5 ml of fasting blood was drawn using BD Vacationer vacuum blood collection tubes (Suzhou BD Medical Devices Co., Ltd.). The following blood lipid parameters were measured using an analyzer (Roche Diagnostics Co., Ltd. (Shanghai)) at baseline and after half a year of evolocumab plus statin therapy: total cholesterol (TC), TG, high-density lipoprotein cholesterol (HDL-c), LDL-c, small dense low-density lipoprotein (sd-LDL), and 1ipoprotein(a) (LP(a)).

### US imaging acquisition

B-mode ultrasonography and CEUS were performed at baseline and after half a year of evolocumab plus statin therapy using a GE Logic E9 ultrasound system (GE Medical Systems Ultrasound and Primary Care Diagnostics; Wauwatosa, WI, USA) equipped with a 9-L probe at a transmission frequency of 9–11 MHz. All procedures were performed by a vascular radiologist (Ju Chen) with 15 years of experience who was blinded to the medical history and blood results of the participants.

Each patient was requested to lie in the supine position and fully expand the neck. The left and right common carotid arteries, extracranial segments of the internal carotid artery and external carotid artery were examined in the transverse and longitudinal views on B-mode images. Plaque was classified as follows: hyperechoic plaque was defined as a ratio of calcified area to total plaque area of greater than 90%; homogeneous echolucent plaque was defined as a plaque with an echogenicity less than that of the surrounding adventitia in >80% of the area, without acoustic shadowing; mixed plaque was defined as plaque containing <90% of the circumferential calcification or with associated echo-dense and anechoic regions that occupied <80% of the plaque area (Staub et al., 2011). Echolucent and mixed plaques were analyzed.

CEUS imaging was performed after the administration of SonoVue (Bracco SpA, Milan, Italy). A 1.6 ml bolus was injected *via* the antecubital vein within 2–3 s, followed by a 5 ml saline flush. Since it has a mean diameter of 2.5 µm (Kern et al., 2004), SonoVue can freely flow in tiny capillaries but does not pass through the vascular bed into the tissue space, making it an ideal intravascular tracer (Ferrara et al., 2000). The image setting was adjusted to maximize the visualization of SonoVue, with a machine index of 0.11–0.12, focusing below the region of interest (ROI), and with maximum signal gain and minimum noise. Cine clips were recorded immediately after injection and lasted for 3 min. All clips were stored for further analysis. The patients were observed for at least 30 min in case any complications occurred.

### US image analysis

The maximal plaque thickness, calcified area and hypoechoic area were measured on the thickest cross-section of plaque. Maximal plaque thickness was measured from the media-adventitia to intima-lumen boundaries. The calcified area and hypoechoic area of the plaque were measured by B-mode ultrasound imaging on the maximum section of the long axis of the plaque by ImageJ software.

Plaques thicker than 1.5 mm were further analyzed by CEUS. First, CEUS imaging was quantitatively evaluated using time-signal intensity curve analysis software (TIC Analysis; TomTec, Germany) on the GE Logic E9. ROIs of similar size and depth were drawn around the margin of the plaque and in the surrounding lumen to obtain the following specific parameters: background intensity (BI), peak intensity (PI), enhanced intensity and area under the curve (AUC) of the plaque and lumen. The AUC reflected the total amount of acoustic developer entering the plaque or lumen within the measured 180 s; the enhanced intensity reflected the peak blood flow intensity during the monitoring period according to the formula: enhanced intensity = PI-BI. To avoid error caused by the background signal during each measurement, we used the standard method for the AUC and enhanced intensity values: AUC (P/L) = AUC in plaque/AUC in lumen; enhanced intensity (P/L) = (PI-BI) in plaque/(PI-BI) in lumen. The contrast agent area reflected the area of vessels in the plaques. We then used ImageJ to calculate the area ratio of contrast agent in the plaque, which was expressed as its ratio to the plaque area.

For each plaque, the neovascularization score was used to semiquantitatively categorize the degree of enhancement as follows: 0 points: no contrast medium in the plaque; 1 point: punctate contrast medium in the plaque; 2 points: between 1 and 3 points; 3 points: linear contrast medium in the plaque (Shah et al., 2007).

Two radiologists each with 10 years of experience who were blinded to the participants’ information independently analyzed the TIC parameter. If any disagreements occurred, another senior radiologist took part in the interpretation until a consensus was reached.

### Statistical analysis

Values are reported as the mean ± standard deviation, where appropriate. The data analysis was performed using SPSS 26.0 (SPSS, Inc., Chicago, IL, USA). The *t* test of paired samples was used to compare parameters at baseline and after half a year of evolocumab plus statin therapy. Unpaired t tests were used for the subgroup analyses. *p* < 0.05 was considered to indicate a statistically significant difference.

## Results

### Baseline characteristics

During the study period, 31 subjects were enrolled for analysis: 20 males with a mean age of 56.95 ± 10.23 years and 11 females with a mean age of 62.00 ± 5.58 years. The number of patients with different risk factors was as follows: 11 patients had diabetes mellitus, 20 patients had hypertension, 6 patients had an active smoking history, 28 patients had coronary artery disease, 3 patients had experienced stroke and 2 patients had peripheral arterial disease. All subjects were diagnosed with atherosclerotic cardiovascular disease (ASCVD), and many types of drugs had been used, such as β-blockers and aspirin. The 31 patients included in the study presented with a total of 33 carotid plaques. The baseline characteristics of the included patients are shown in Table 1. Table 2 shows the changes in heart rate, blood pressure, blood lipid profiles and biochemistry indices pre- and posttherapy.

**TABLE 2:** Comparison of heart rate, blood pressure, blood lipid and biochemical indexes.

Item	Baseline	Half year	*p*-value
Heart rate (beats/minute)	74.39 ± 7.89	72.55 ± 7.60	0.250
Systolic pressure (mmHg)	133.35 ± 15.47	132.09 ± 14.61	0.686
Diastolic pressure (mmHg)	75.71 ± 10.69	77.61 ± 8.50	0.497
LP(a) (mg/L)	202.26 ± 347.42	147.17 ± 243.67	0.274
TC (mmol/L)	3.84 ± 0.99	2.55 ± 0.72	0.000
TG (mmol/L)	1.66 ± 0.83	1.48 ± 1.63	0.560
HDL-c (mmol/L)	1.08 ± 0.34	1.30 ± 0.26	0.000
LDL-c (mmol/L)	2.23 ± 0.75	0.95 ± 0.63	0.000
sd-LDL (mmol/L)	0.69 ± 0.32	0.33 ± 0.25	0.000
ALT (U/L)	31.28 ± 32.03	22.32 ± 12.97	0.055
AST (U/L)	27.09 ± 39.94	20.79 ± 10.14	0.300
ALP(U/L)	68.30 ± 15.60	69.88 ± 14.37	0.422
TBIL (μmol/L)	10.74 ± 3.78	11.45 ± 4.33	0.262
Albumin (g/L)	40.93 ± 8.25	44.48 ± 6.80	0.065
Prealbumin (mg/L)	258.97 ± 50.05	270.84 ± 47.35	0.259
Glucose (mmol/L)	6.22 ± 1.41	6.31 ± 0.79	0.723
Urea (mmol/L)	5.68 ± 1.96	5.86 ± 1.94	0.353
Creatinine (μmol/L)	74.23 ± 24.35	74.53 ± 20.49	0.686

All data are presented as the mean ± SD; ALT, alanine aminotransferase; AST, aspartate aminotransferase; ALP, alkaline phosphatase; TBIL, total bilirubin; TC, total cholesterol; TG, triglyceride; HDL-c, high-density lipoprotein cholesterol; LDL-c, low-density lipoprotein cholesterol; LP(a), lipoprotein(a); sd-LDL, small dense low-density lipoprotein.

### Effect of evolocumab plus statin therapy on blood lipid levels

Consistent with several other large studies, the lipid profiles changed significantly after evolocumab plus statin treatment. Compared with baseline, LDL-c decreased by approximately 57% (2.23 ± 0.75 mmol/L vs. 0.95 ± 0.63 mmol/L, *p* < 0.001); TC decreased by approximately 34% (3.84 ± 0.99 mmol/L vs. 2.55 ± 0.72 mmol/L, *p* < 0.001); TG decreased by approximately 11% (1.66 ± 0.83 mmol/L vs. 1.48 ± 1.63 mmol/L, *p* = 0.560); lipoprotein(a) [LP(a)] decreased by approximately 27% (202.26 ± 347.42 mg/L vs. 147.17 ± 243.67 mg/L, *p* = 0.274); sd-LDL decreased by approximately 52% (0.69 ± 0.32 mmol/L vs. 0.33 ± 0.25 mmol/L, *p* < 0.001); and HDL-c increased by approximately 20% (1.08 ± 0.34 mmol/L vs. 1.30 ± 0.26 mmol/L, *p* < 0.001) (Table 2).

### Effect of evolocumab plus statin therapy on B-mode US features

The most commonly used evaluation indicators for plaques are the maximum length, thickness and cross-sectional area of the plaque’s long axis. Compared with baseline, the length of the plaques decreased from 16.97 ± 10.75 mm to 16.80 ± 10.77 mm (*p* = 0.002), and the thickness of the plaques decreased from 2.81 ± 1.08 mm to 2.73 ± 1.08 mm (*p* = 0.002). These changes were statistically significant.

The hypoechoic area of the plaque/area of plaque was reduced (from 0.037 ± 0.062 to 0.028 ± 0.052; *p* = 0.015). The plaque area, calcified plaque area/plaque area, and lumen cross-sectional area changed little (*p* = 0.222, *p* = 0.264 and *p* = 0.700, respectively) (Table 3).

**TABLE 3:** The changes of ultrasound parameters.

Variable	Baseline	Half year	*p*-value
Contrast-Enhanced Ultrasound			
AUC(P/L)	0.27 ± 0.13	0.19 ± 0.11	0.000
Enhanced Intensity (plaque)	8.64 ± 3.72	7.48 ± 3.75	0.076
Enhanced Intensity (lumen)	23.38 ± 2.34	23.80 ± 3.28	0.537
Intensity ratio (P/L)	0.37 ± 0.16	0.31 ± 0.14	0.009
Agent area in plaque/area of plaque	19.20 ± 13.23	12.66 ± 9.59	0.003
Neovascularization score	2.64 ± 0.54	2.06 ± 0.86	0.000
B Mode Ultrasound			
Lumen cross-sectional area (mm^2^) ^&^	31.25 ± 16.61	31.42 ± 16.82	0.700
The area of plaque (mm^2^)	44.00 ± 59.16	43.04 ± 57.64	0.222
The thickness of plaque (mm)	2.81 ± 1.08	2.73 ± 1.08	0.001
The length of plaque (mm)	16.97 ± 10.75	16.80 ± 10.77	0.002
Calcified area in plaque/area of plaque	0.10 ± 0.13	0.11 ± 0.12	0.264
Hypoechoic area in plaque/area of plaque	0.039 ± 0.063	0.030 ± 0.054	0.015

All data are presented as the means ± SD.; AUC, area under curve; P/L, plaque/lumen; enhanced intensity = peak intensity—baseline intensity; intensity ratio = enhanced intensity (plaque)/enhanced intensity (lumen) &: measure the lumen cross-sectional area at the point where the plaque is the thickest.

### Effect of evolocumab plus statin therapy on CEUS parameters

**FIGURE 2:**
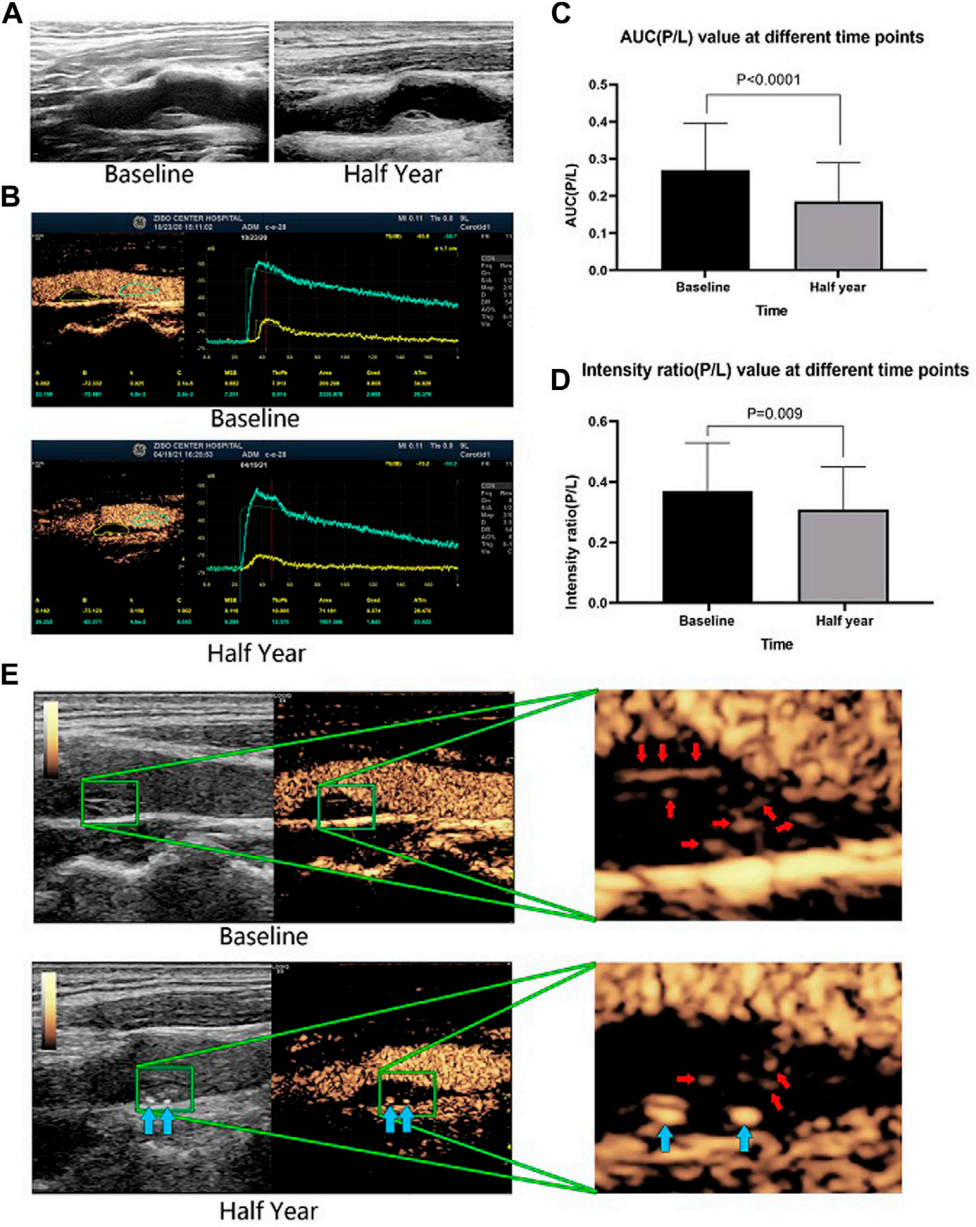
A representative patient who had a large plaque in the common carotid artery that was identified by B-mode ultrasound. The plaque morphology changed little between baseline and after half a year of therapy **(A)**. CEUS revealed that the contrast agent area and microvessel density were obviously reduced after half a year of evolocumab plus statin treatment **(B)**. The AUC (P/L) and the enhanced intensity (P/L) decreased significantly after half a year of treatment **(E)**. Red arrows indicate vessels in the plaque, and blue arrows indicate calcified plaques. The changes in AUC (P/L) and enhanced intensity (P/L) of all included patients before and after half a year of treatment **(C**,**D)**.

The CEUS results demonstrated that AUC (P/L), which represents the total amount of contrast agent in the intraplaque microvessels during 180 s of monitoring, was significantly reduced from 0.27 ± 0.13 at baseline to 0.19 ± 0.11 after half a year of evolocumab plus statin therapy (*p* < 0.001). The intensity ratio (P/L), which represents the peak flow velocity of contrast agent into the intraplaque microvessels, was reduced from 0.37 ± 0.16 at baseline to 0.31 ± 0.14 after half a year of therapy (*p* = 0.009). The contrast agent area in plaque/area of plaque, which represents the density of microvessels in a plaque, also decreased, from 19.20 ± 13.23 at baseline to 12.66 ± 9.59 after half a year of therapy (*p* = 0.003). The neovascularization score was also significantly reduced from 2.64 ± 0.54 to 2.06 ± 0.86 (*p* < 0.001) (Table 3 and Figure 2).

Figure 2 shows a representative patient who had a large plaque in the common carotid artery that was identified by B-mode ultrasound. Although the plaque morphology changed little (Figure 2A), CEUS revealed that the contrast agent area and microvessel density were obviously reduced (Figure 2B), and the AUC (P/L) and the intensity ratio (P/L) had decreased significantly (Figure 2C) after half a year of evolocumab plus statin treatment.

### Influence of statins on the endpoints

As a single-arm study, the largest limitation of this study was that there was no statin control group. However, statins are certain to have an impact on the outcomes. To limit the effect of statins as confounding factors, the patients were initially asked to start statin therapy for at least 1 month. We then evaluated the influence of different durations of statin use on the outcomes. Subgroup analysis was performed according to the duration of statin administration before the addition of evolocumab (<6 months and ≥6 months). The AUC and intensity ratio values of different durations of statin administration were compared at baseline and 6 months, respectively. However, they were not significantly different (*p* > 0.05) Table 4.

**TABLE 4:** Subgroup analysis based on statin duration.

Variable	<6 months	≥6 months	*p*-value
AUC(P/L) (baseline)	0.31	0.25	0.173
AUC(P/L) (half year)	0.21	0.17	0.328
AUC(P/L) rate	0.33	0.24	0.449
Intensity ratio (P/L) (baseline)	0.40	0.35	0.381
Intensity ratio (P/L) (half year)	0.34	0.28	0.226
Intensity ratio (P/L)[(baseline-half year)/baseline]	0.13	0.13	0.974

AUC (P/L), area under curve of plaque/area under curve of lumen; AUC(P/L) rate, [AUC(P/L) (baseline)- AUC(P/L) (half year)]/AUC(P/L) (baseline); Intensity ratio (P/L), the difference of peak intensity and baseline intensity in the plaque/the difference of peak intensity and baseline intensity in the lumen.

## Discussion

In this study, evolocumab plus statins significantly reduced the LDL-c levels in patients. Furthermore, the decreased CEUS parameters indicated that the plaques were stabilized by inhibiting IPN, which might reduce the occurrence of cardiovascular events.

Previous clinical studies (Scandinavian Simvastatin Survival Study Group, 1994; Colhoun et al., 2004; Heart Protection Study Collaborative Group, 2002; Long-Term Intervention with Pravastatin in Ischaemic Disease (LIPID) Study Group, 1998) have confirmed that statins can significantly reduce cardiovascular mortality and all-cause mortality in patients with atherosclerosis; therefore, statins have been seen as the cornerstone treatment for atherosclerotic diseases. However, statins have their own limitations. First, if the statin dose is doubled, its lipid-lowering effect is increased by only 6% (Zhao et al., 2014), which makes it difficult for many patients to achieve the LDL-c goal with statins alone. Second, some patients cannot tolerate statins (Reiner, 2018); this has been particularly identified in Chinese patients who are administered high-dose statins (Group et al., 2014).

Evolocumab provides a new regimen for these patients. It can reduce LDL-c levels by specifically binding PCSK9. The GAUSS-3 study (Goal Achievement after Utilizing an anti-PCK9 antibody in Statin Intolerant Subjects-3) confirmed that evolocumab was an effective alternative to reduce LDL-c in patients at high risk of ASCVD who had statin intolerance or familial hypercholesterolemia (Nissen et al., 2016). The FOURIER study proved that the addition of evolocumab to statin therapy could further reduce LDL-c by 59% and reduced the relative risk of major cardiovascular events by 15–20%. Further analysis showed that there was no lower limit for the reduction of LDL-c by evolocumab (Sabatine et al., 2017).

In our study, compared with baseline, the LDL-c level decreased by approximately 57% after a half year of evolocumab treatment. The change trends of other blood lipid indices, such as TC and HDL-c, were similar to the results of the FOURIER study. Sd-LDL is a subtype of LDL-c, and its metabolic pathway is the same as that of LDL-c. When LDL-c was reduced by evolocumab, the sd-LDL level also decreased. A previous study confirmed that sd-LDL was the most atherogenic lipoprotein parameter (Ikezaki et al., 2021). Theoretically, evolocumab can prevent, delay and even reverse the development of plaques.

One important feature of plaque vulnerability is IPN. Cui et al. (Cui et al., 2021) demonstrated that carotid IPN was an independent predictor of future vascular events in patients with recent ischemic stroke. Song et al. (Song et al., 2020) also showed that IPN was a risk factor for the occurrence of the clinical symptoms of stroke. Recent clinical studies have shown that statins can inhibit IPN (Xu et al., 2018; Du et al., 2019). In our study, the AUC (P/L), intensity ratio (P/L), agent area in plaque/area of plaque, and neovascularization score indicated that the amount of IPN had decreased. Our observations indicate that evolocumab inhibits IPN, similar to statins, and this occurs for several reasons. First, the GLAGOV study confirmed that compared with placebo, evolocumab reversed atherosclerotic plaque and reduced the plaque volume (Nicholls et al., 2016). This meant that there was a corresponding reduction in the amount of nutrients and oxygen required by the plaque, so the number of new blood vessels in the plaque was correspondingly reduced. Second, an important mechanism of IPN is vascular inflammation (Chistiakov et al., 2017). Current studies have demonstrated that ecolocumab has anti-inflammatory effects, so it might also reduce IPN in this way (Hoogeveen et al., 2019; Momtazi-Borojeni et al., 2019; Vlachopoulos et al., 2019). Third, evolocumab improves the blood lipid profile by reducing TC, LDL-c, sd-LDL, LP(a), and TG and increasing HDL-c. Improvement of the blood lipid profile itself will also inhibit IPN (Wang et al., 2021).

Other than CEUS, the MRA, positron emission tomography (PET) and computerized tomography angiography (CTA) techniques can also be used to assess angiogenesis within plaques. However, CEUS has some advantages; it is non-invasive, inexpensive, and has high temporal and spatial resolution. Most importantly, it has been confirmed that its results are well correlated with histopathological results (Saito et al., 2014; Huang et al., 2016) and it can be used predict future vascular events in patients (Song et al., 2020; Cui et al., 2021). Using CEUS to assess the stability of carotid atherosclerotic plaques and guide medication decision-making will help to reduce the incidence of ischemic stroke and improve patients’ health. Therefore, the clinical use of this technique should be promoted.

An important limitation of the present study was that it was a single-arm study with no control group. To minimize the confounding influence of background statins on the study results, we performed a subgroup analysis. The subgroup analysis indicated that there was no significant difference in the indicators of neovascularization in the plaque between patients who had used statins for less than 6 months before enrollment and patients who had used statins for more than 6 months. Xu et al. reported that IPN changes could occur after 1 year of statin treatment (Xu et al., 2018), while Du et al. found IPN changes after 3 months of rosuvastatin treatment (Du et al., 2019). Therefore, our study demonstrated that even in patients with a well-documented statin background, the use of evolocumab could further reduce IPN. Nevertheless, larger randomized controlled trials are needed to confirm the relationship between evolocumab and IPN.

In conclusion, our findings suggest that evolocumab effectively reduced LDL-c and improved the blood lipid profile. Evolocumab therapy significantly reduced IPN and thereby stabilized plaques.

## Data Availability

The original contributions presented in the study are included in the article/supplementary material, further inquiries can be directed to the corresponding authors.
